# Genome-Wide Analysis Reveals Extensive Changes in LncRNAs during Skeletal Muscle Development in Hu Sheep

**DOI:** 10.3390/genes8080191

**Published:** 2017-08-01

**Authors:** Caifang Ren, Mingtian Deng, Yixuan Fan, Hua Yang, Guomin Zhang, Xu Feng, Fengzhe Li, Dan Wang, Feng Wang, Yanli Zhang

**Affiliations:** 1 Jiangsu Engineering Technology Research Center of Mutton Sheep and Goat Industry, Nanjing Agricultural University, Nanjing 210095, China; rencaifang@hotmail.com (C.R.); dengmti@hotmail.com (M.D.); fanyixuan@njau.edu.cn (Y.F.); 2015105031@njau.edu.cn (H.Y.); zhangguomin@njau.edu.cn (G.Z.); fox120804@outlook.com (X.F.); lfz162518@live.com (F.L.); wangdan@njau.edu.cn (D.W.); caeet@njau.edu.cn (F.W.); 2 Jiangsu Livestock Embryo Engineering Laboratory, Nanjing Agricultural University, Nanjing 210095, China

**Keywords:** lncRNA, muscle growth, RNA-Sequencing, sheep

## Abstract

As an important type of noncoding RNA molecules, long non-coding RNAs (lncRNAs) act as versatile players in various biological processes. However, little is known about lncRNA regulators during sheep muscle growth. To explore functional lncRNAs during sheep muscle growth, we systematically investigated lncRNAs using strand-specific Ribo-Zero RNA sequencing at three key developmental stages in Hu sheep. A total of 6924 lncRNAs were obtained, and the differentially expressed lncRNAs and genes were screened from (control vs. experiment) fetus vs. lamb, lamb vs. adult, and fetus vs. adult comparisons, respectively. The quantitative real-time polymerase chain reaction (qRT-PCR) analysis results correlated well with the sequencing data. Moreover, functional annotation analysis based on the Gene Ontology (GO) and Kyoto encyclopedia of genes and genomes (KEGG) databases showed that the target genes of the differentially expressed lncRNAs were significantly enriched in organ morphogenesis, skeletal system development as well as response to stimulus and some other terms related to muscle. Furthermore, a co-expression network of the differentially expressed target genes and lncRNAs was constructed and well-known muscle growth regulators such as retrotransposon-like 1 and Junctophilin-2 were included. Finally, we investigated the expression profiles of seven lncRNAs and their target genes, and found that they played vital roles in muscle growth. This study extends the sheep muscle lncRNA database and provides novel candidate regulators for future genetic and molecular studies on sheep muscle growth, which is helpful for optimizing the production of mutton.

## 1. Introduction

Mutton is becoming a popular meat due to its high-protein, low-fat, and low-cholesterol features. Improving muscle growth in sheep may increase mutton productivity. Skeletal muscle growth is a stepwise, progressive process of differentiation, specification and maturation, which is precisely orchestrated by molecular regulation networks. In the fetal stage, myogenesis is mainly driven by the myocyte enhancer factor 2 (MEF2) [1] family members and muscle regulatory factors [2]. During the first few weeks of postnatal life, the maturation of the internal architecture of myofibers makes them drive more complicated functions [3]. Adult muscle is completely mature, with multiple roles depending on the predetermined myofiber type identity. Several key markers of important phenotypic traits of muscle growth, such as myostatin [4] and callipyge (CLPG) [5], have been characterized. However, previous studies on the growth of sheep skeletal muscle have primarily focused on protein-coding genes, whereas the vast majority of the genome (demonstrated in humans) bears noncoding sequences [6]. As an important type of noncoding RNA molecules, long noncoding RNAs (lncRNAs) act as versatile players in various biological processes. 

Accumulating studies have shown that some functional lncRNAs serve as novel regulators in muscle biology. During myogenesis, lnc-MD1, one of the first discovered lncRNAs, upregulates the expression of MEF2C and mastermind like transcriptional coactivator 1 by competitive binding with microRNAs such as miR-135 and miR-133, and consequently activates late differentiation [7]. Another known lncRNA, developmental pluripotency-associated 2 (Dppa2) Upstream binding Muscle lncRNA, silences its neighbor gene through interacting and recruiting multiple DNA methyltransferases to its promoter regions [8]. During the course of adult fast-type myofiber specialization, long intergenic non-coding RNA (lincRNA), lincMYH is specifically expressed in the nuclei of fast-type myofibers and prevents slow-type gene expression, thus locking their phenotype [9]. Moreover, lncRNA might encode hidden functional polypeptide, and therefore is involved in the regulation of muscle performance [10,11,12]. Additionally, two newly identified lncRNAs, linc-RAM [13] and lnc-mg [14], have extended the functional lncRNA database for muscle differentiation and regeneration. These studies indicate the important roles of lncRNAs in muscle biology. 

However, limited transcriptomic research on sheep lncRNAs related to muscle has been carried out [15,16], especially Hu Sheep, a famous Chinese endemic species bred for meat and skin. The expression pattern and potential values of lncRNAs in the development of sheep skeletal muscle remain largely unknown. It is necessary to understand the dynamics of the sheep muscle transcriptome at different stages as the proliferation of sheep myofibers proceeds before or around 100 days of gestation, and then myofibers grow to fuse together and hypertrophy [17]. Thus, we investigated the expression pattern and potential roles of lncRNAs in sheep muscle at three key developmental stages (110-day fetus, five-day-old lamb, and two-year-old adult). The target genes of the differentially expressed lncRNAs and the differentially expressed genes (DEGs) were examined. Most importantly, potential lncRNA regulators of sheep muscle growth were predicted by the lncRNA-gene network. This study expands the sheep muscle lncRNA catalogue and provides candidate regulators of sheep myofiber growth at the transcriptional level.

## 2. Materials and Methods 

### 2.1. Animals and Sample Collection 

All experimental procedures involving the use of animals were approved and carried out in accordance with the relevant guidelines set by the Ethics Committee of Nanjing Agricultural University, China (Approval ID: SYXK2011-0036). 

Sheep used in this experiment were raised under the same condition with natural light and free access to food and water at Taizhou Hailun Sheep Industry Co., Ltd. (Taizhou, China). LD muscle samples at a pre-determined site (between the 12th and 13th thoracic vertebrae) were collected from nine Hu rams at fetus, lamb, and adult stages (*n* = 3 at each stage) and immediately frozen in liquid nitrogen for total RNA extraction after harvesting. 

### 2.2. Library Preparation

Total RNA was isolated with TRIzol reagent (Invitrogen, Carlsbad, CA, USA) and treated with DNase I (Qiagen, Beijing, China). The purified RNA was further monitored on 1.5% agarose gels to ensure no genomic DNA contamination. RNA integrity was assessed using the RNA Nano 6000 Assay Kit of the Agilent Bioanalyzer 2100 System (Agilent Technologies, Santa Clara, CA, USA) and equal amounts of RNA samples (1.5 μg RNA per sample) were used to prepare Ribo-Zero RNA-Sequencing (RNA-Seq) library. Ribosomal RNA (rRNA) was removed using the Ribo-Zero rRNA Removal Kit (Epicentre, Madison, WI, USA). 

The strand-specific sequencing libraries were separately prepared using NEBNextR UltraTM Directional RNA Library Prep Kit for IlluminaR (NEB, Ipswich, MA, USA) according to the manufacturer’s instructions. Specifically, the rRNA-depleted RNA samples were fragmented using divalent cations under elevated temperature in 5X NEBNext First Strand Synthesis Reaction Buffer. The synthesis of first-strand complementary DNA (cDNA) was performed using random hexamer primers and reverse transcriptase, and the double-stranded cDNA was subsequently synthesized using Polymerase I, RNase H, dATP, dUTP, dCTP and dGTP. Remaining overhangs were converted into blunt ends via exonuclease/polymerase activities. Following adenylation of the 3’ ends of DNA fragments and adaptor ligation, 150–200 base pairs (bp) cDNA fragments were isolated with AMPure XP Beads (Beckman Coulter, Beverly, MA, USA). The strands containing U base were removed using USER Enzyme (NEB). After enrichment with Polymerase Chain Reaction (PCR), in which the Index (X) Primer was used, the final cDNA libraries were created and assessed with the Agilent Bioanalyzer 2100 and quantitative real-time PCR (qRT-PCR). 

### 2.3. Clustering, Sequencing, and Transcriptome Assembly

Clusters were generated on the acBot Cluster Generation System using TruSeq PE Cluster Kit v3-cBot-HS (Illumia) according to the manufacturer’s instructions and further sequenced on an Illumina Hiseq platform. Quality control of the RNA-Seq reads was performed using FastQC (http://www.bioinformatics.babraham.ac.uk/projects/fastqc/). Clean reads were obtained by removing reads containing adapter and ploy-N as well as low quality reads from raw data. At the same time, the Q30, GC content and sequence duplication level of the clean data were calculated. Reads that passed the quality control were then mapped to the *Ovis aries* reference genome (Oar_v3.1). Based on it, the transcripts were assembled with Cufflinks (version 2.1.1) [18] and Scripture [19]. 

### 2.4. Prediction of Multiple-Exon Long Non-Coding RNA and Protein-Coding RNA

After annotation, the unknown transcripts were used to screen for lncRNA candidates. Transcripts smaller than 200 nucleotides or that have single exon were discarded firstly. Based on the length of open reading frame, homology with known proteins, protein domains and their coding potential, the Coding Potential Calculator [20], the Coding-Non-Coding Index [21], the Protein Families Database [22] and the Coding-Potential Assessment Tool [23], which have the power to sort lncRNAs from putative protein-coding RNAs were combined to screen the lncRNAs. The transcripts from the intersection of the four methods with fragments per kilo base of exon per million fragments mapped (FPKM) greater than 0.1 were kept and predicted to be lncRNA transcripts. 

### 2.5. Differential Expression Analysis and Quantitative Real-Time Polymerase Chain Reaction Validation 

Transcript abundance was measured by FPKM using Cuffdiff (version 2.1.1) [24]. The FPKMs of the protein-coding genes in each sample were computed by summing the FPKMs of the transcripts in each gene group. Differential expression analysis of the groups was performed using the DEGseq packages (1.10.1) [25]. LncRNAs or protein-coding genes with a false discovery rate (FDR) <5% and an absolute value of log2 (fold change) >1 were assigned as differentially expressed. 

For the qRT-PCR analysis, 1 μg of total RNA was reverse transcribed using the RT reagent Kits with gDNA Eraser (Takara, Dalian, China) according to the manufacturer’s protocol. QRT-PCR was performed on a StepOnePlus Real-Time PCR System (Life Technologies, USA) according to standard methods using Fast Start Universal SYBR Green Master (ROX) (Roche, Mannheim, Germany). Five differentially expressed lncRNAs and six DEGs were chosen for qRT-PCR validation, including a muscle structural gene myosin heavy chain 7 (MYH7) and myogenin (MYOG). Comparative quantification of each gene was normalized to hypoxanthine phosphoribosyltransferase 1 using the 2^−ΔΔCt^ method. All experiments were performed in triplicate. 

### 2.6. Target Gene Prediction and Functional Annotation Analysis 

The target genes of the differentially expressed lncRNAs were predicted through two methods. For each lncRNA locus, the 100 kb upstream and downstream protein-coding genes (without overlap) were firstly identified as cis-acting target genes. Then the genes that overlapped with the lncRNAs predicted by Lnctar (http://www.cuilab.cn/lnctar) were selected as trans-acting target genes. Based on the target genes, lncRNAs could be enriched in the candidate biological processes. 

To analyze the main function of the lncRNAs, the target genes and DEGs were annotated through NCBI non-redundant protein database (Nr), the Gene Ontology (GO) and Kyoto encyclopedia of genes and genomes (KEGG) and Clusters of Orthologous Groups of proteins (COG). GO terms with KS ≤ 0.05 and pathways with corrected *p* ≤ 0.05 were defined as significantly enriched. 

### 2.7. Construction and Verification of the LncRNA-Gene Co-Expression Network

To further explore the interactions between the lncRNAs and their target genes, the differentially expressed target genes of the differentially expressed lncRNAs from each comparison were further screened. Based on their FPKMs, the co-expression correlations were calculated. Then the lncRNA-gene co-regulated pairs (COR > 0.8 and *p* < 0.05) and their relationships were integrated to construct the lncRNA-gene co-expression network. The lncRNA-gene pairs associated with muscle growth were sorted with reference to their GO enriched terms with key words, including myoblast, muscle development and skeletal development. Finally, we analyzed the expression of six co-expressed lncRNAs and their corresponding target genes during muscle development using qRT-PCR for better verification of the RNA-Seq data. 

### 2.8. Statistical Analysis

All experiments were repeated at least three times. Results of the qRT-PCR data were presented as the mean ± standard error (SEM). Statistically analyses were performed using the software SPSS 18.0 (SPSS Inc., Chicago, IL, USA). Differences were regarded as significant at *p* < 0.05.

## 3. Results

### 3.1. Read Mapping and Transcript Assembly 

The mean GC content of the 9 libraries was 54.39% and the Q30 of each sample was not less than 91.71%, suggesting that the sequencing data were highly reliable. On average, 65,578,070, 65,591,958, and 71,241,551 mapped reads of the fetus, lamb, and adult stages, respectively, were obtained from the clean data, and more than 92% were uniquely mapped to the *O. aries* reference genome. Moreover, location analysis of these mapped reads revealed an average of 45.1% reads were mapped to exonic regions in the fetus stage while an increase was observed in the later stages (53.8% in lamb and 53.7% in adult). In contrast, the proportions of intronic and intergenic reads were lower in the lamb and adult muscle compared with those in the fetus muscle (Table 1). 

### 3.2. Identification and Characterization of Long Non-Coding RNA in Sheep Muscle 

**Figure 1:**
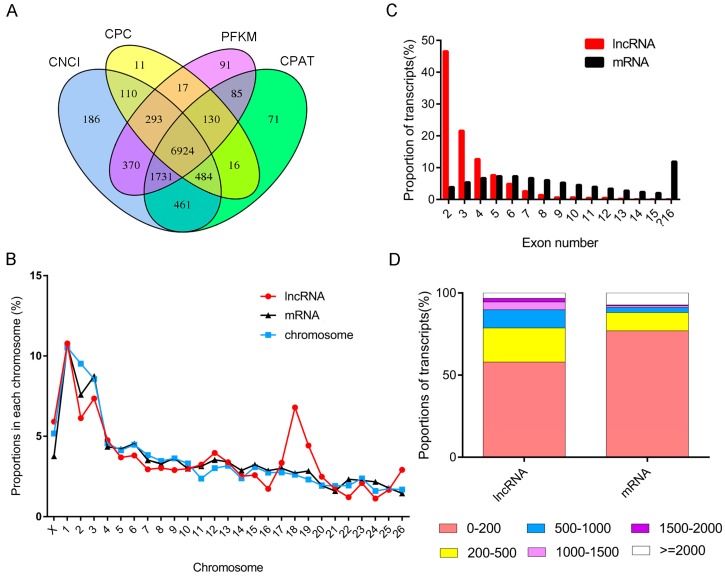
The features of sheep muscle long non-coding RNA (lncRNAs) and mRNAs. (**A**) Venn diagram of noncoding transcripts from the Coding Potential Calculator (CPC) [20], the Coding-Non-Coding Index (CNCI) [21], the Protein Families Database (PFAM) [22] and the Coding-Potential Assessment Tool (CPAT). (**B**) Distribution of lncRNAs and mRNAs along each chromosome. The red and black lines indicate the proportion of lncRNAs and protein-coding genes respectively, the blue line indicates the size proportion of the corresponding chromosome in the genome. (**C**) Exon numbers per transcript of sheep muscle lncRNAs. (**D**) Exon size distribution of sheep muscle lncRNAs.

To study the basic features of lncRNAs in sheep muscle, the lncRNAs were identified and compared with mRNAs. The intersection of the Coding Potential Calculator, Coding-Non-Coding Index, the Protein Families Database and Coding-Potential Assessment Tool results finally yielded 6924 lncRNA transcripts including the identified conservative lncRNA, muscle differentiation (lncMD) [26] (Figure 1A). The lncRNA transcripts were classified as 4606 (66.5%) lincRNAs, 1131 (16.3%) intronic lncRNAs and 1187 (17.1%) anti-sense lncRNAs. Similar to mRNAs, lncRNA transcripts were distributed widely in 26 autosomes and the X-chromosome, but no lncRNA was found in the mitochondria. In addition, the proportions of the lncRNAs in several chromosomes were inconsistent with the chromosome size proportion, especially chromosome 18, suggesting its corresponding lncRNAs might play particular roles in this study (Figure 1B). Comparison of the exon features of the lncRNAs and mRNAs showed that most lncRNAs contained 2-5 exons per transcript (about 3.3, on average), which was fewer than the mRNAs (about 7.9, on average; Figure 1C). Moreover, most lncRNAs contained two exons; while two-exon mRNAs accounted for only 3.9% of the total mRNAs, the number was apparently lower than that of lncRNAs. However, the average size of the exons in the lncRNAs was relatively longer than that of the mRNAs, which was mostly within 200 bp (Figure 1D). 

**Figure 2:**
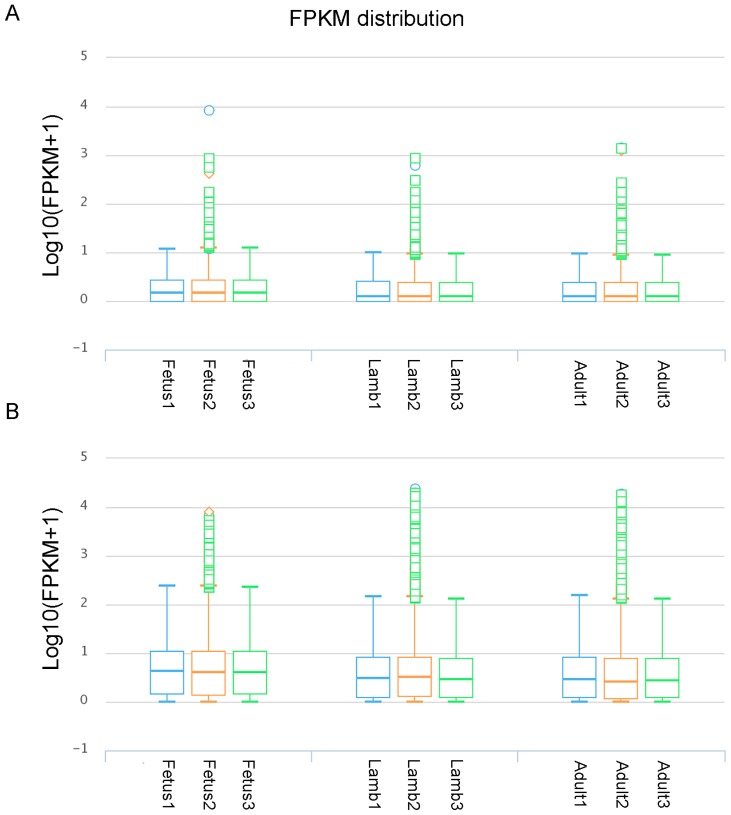
The fragments per kilo base of exon per million fragments mapped (FPKM) distribution of lncRNAs and protein-coding genes at the fetus, lamb, and adult stages during sheep muscle growth, respectively. (**A**) The FPKM distribution of all identified lncRNAs of sheep muscle at three stages. (**B**) The FPKM distribution of protein-coding genes of sheep muscle at three stages.

**Figure 3:**
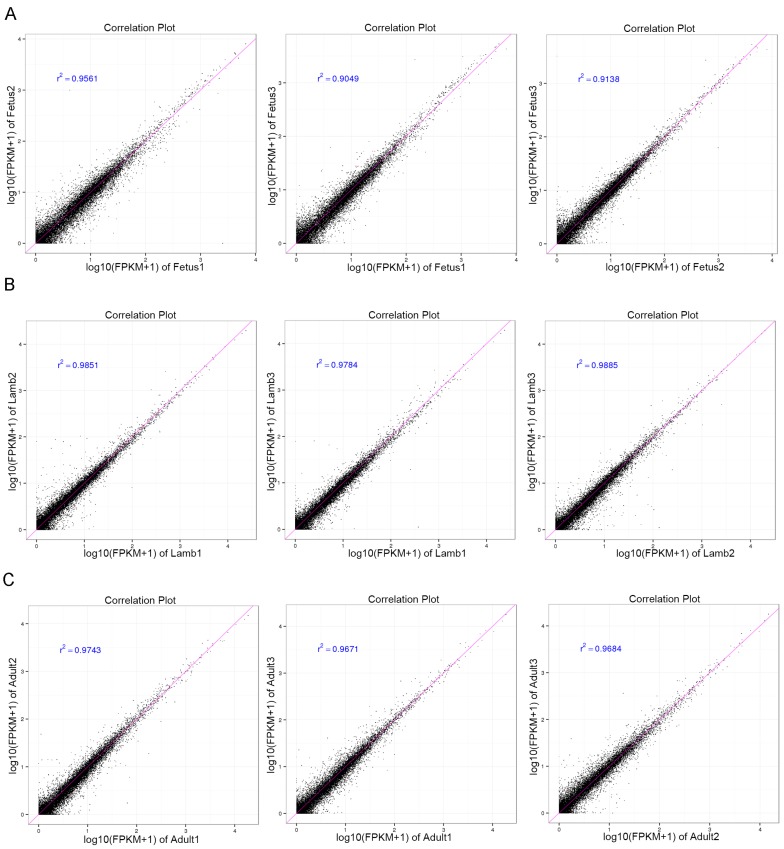
The correlation coefficients analysis between replicates in each group. (**A**) The correlation coefficients between fetus 1, fetus 2, and fetus 3. (**B**) The correlation coefficients between lamb 1, lamb 2, and lamb 3. (**C**) The correlation coefficients between adult 1, adult 2, and adult 3.

In accordance with the protein-coding genes, the global expression trend of lncRNAs during muscle growth was similar in the same stage, and the average expression level of lncRNAs was lower than that of protein-coding genes (Figure 2A,B). The correlation coefficients between replicates in each group were analyzed. The results showed the correlation between replicates in each group was high (Figure 3A–C). In addition, among the 6924 expressed lncRNA transcripts, 33.03% were specifically expressed in one stage while the proportion was considerably lower in protein-coding genes (4.0%), which implied a specific role and dynamic feature of the lncRNAs. Moreover, the number of stage-specific lncRNAs in the fetus (1042) group was higher than that in the lamb (626) and adult (619) groups, suggesting the importance of lncRNAs in the early developmental stage.

### 3.3. Differential Expression Analysis and Target Gene Prediction

**Figure 4:**
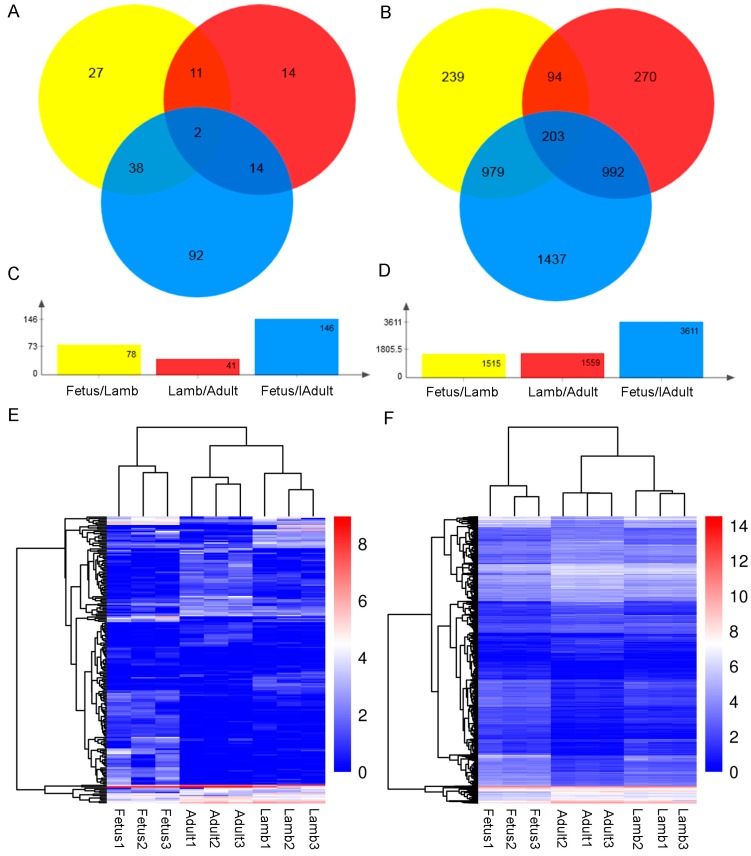
The number of differentially expressed lncRNAs and differentially expressed genes (DEGs) in (control/experiment) fetus/lamb, lamb/adult and fetus/adult comparisons. (**A**) Venn diagram of the number of differentially expressed lncRNAs among different comparisons. (**B**) Venn diagram of the number of DEGs among different comparisons. (**C**) Total number of differentially expressed lncRNAs in each comparison. (**D**) Total number of DEGs in each comparison. (**E**) The hierarchical cluster of differentially expressed lncRNAs. (**F**) The hierarchical cluster of DEGs.

Pairwise comparison between the three stage groups revealed 27, 14 and 92 lncRNAs as well as 239, 270 and 1437 genes were exclusively differentially expressed in the (control vs. experiment) fetus vs. lamb, lamb vs. adult and fetus vs. adult comparisons, respectively (|log2FC| > 1, FDR < 0.05). Notably, lamb vs. adult had the fewest differentially expressed lncRNAs and the number of DEGs in fetus vs. lamb was almost as high as lamb vs. adult, which indicated the transcriptional difference between prenatal and postnatal muscle was large although the time difference was only 1 month (Figure 4A–D). For the lncRNAs, 36 upregulated and 42 downregulated lncRNAs in fetus vs. lamb, 13 upregulated and 28 downregulated lncRNAs in lamb vs. adult and 68 upregulated and 78 downregulated lncRNAs in fetus vs. adult were found. Regarding the DEGs, 1028 upregulated and 487 downregulated in fetus vs. lamb, 659 upregulated and 900 downregulated in lamb vs. adult and 1862 upregulated and 1749 downregulated genes in fetus vs. adult were obtained. The hierarchical cluster of the differentially expressed lncRNAs and DEGs also showed that the expression patterns of the lamb and adult groups were similar but were different from that of the fetus group (Figure 4E,F). 

**Figure 5:**
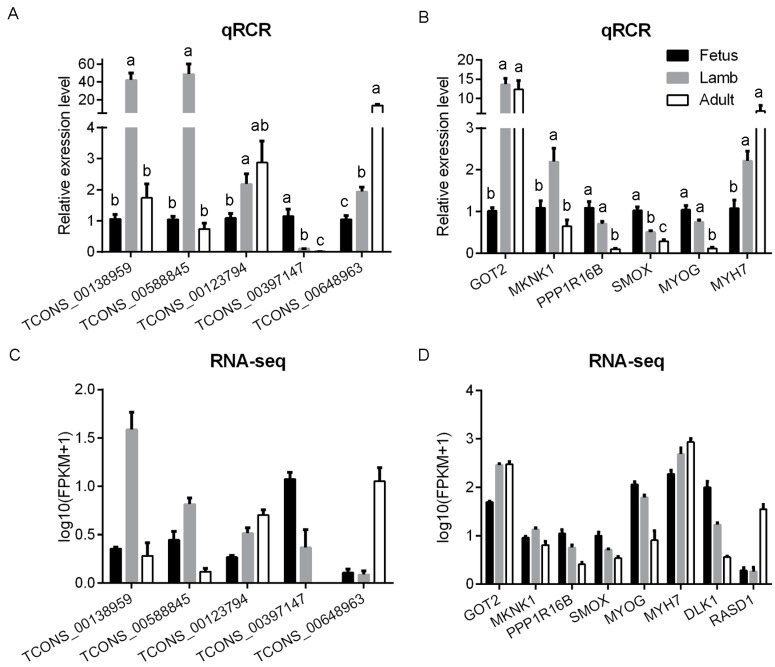
The verification of expression level of differentially expressed transcripts at fetus, lamb and adult stages measured by qRT-PCR and RNA-Seq, respectively. (**A**) The relative expression level of differentially expressed lncRNAs in different stages determined by qRT-PCR. (**B**) The relative expression level of DEGs, including GOT2 (glutamic-oxaloacetic transaminase 2), MKNK1 (MAP kinase interacting serine/threonine kinase 1), PPP1R16B (protein phosphatase 1 regulatory subunit 16B), SMOX (spermine oxidase), MYOG (myogenin) and MYH7 (myosin heavy chain 7) in different stages determined by qRT-PCR. (**C**) The relative expression level of the differentially expressed lncRNAs in different stages determined by RNA-Seq, respectively. (**D**) The relative expression level of DEGs in different stages determined by RNA-Seq, respectively. The expression of delta-like1 homologue gene (DLK1, which is enriched in fetal muscle) and ras related dexamethasone induced 1 (RASD1, which is enriched in lamb muscle at 12 weeks of age [28]) were also showed. The relative expression level of the differentially expressed lncRNAs and DEGs in muscle was determined by qRT-PCR and normalized to the expression of hypoxanthine phosphoribosyltransferase 1 (HPRT1). The qRT-PCR data were represented as the mean ± SEM of three biological and technical replicates. Columns with different letters are significantly different at *p* < 0.05. The RNA-Seq data represented the log10 (FPKM+1) of each lncRNA or gene.

To further evaluate the results of the RNA sequencing, MYOG (a gene associated with the late differentiation of muscle cells that was enriched in fetal muscle [27]), MYH7 (a muscle structural gene, the expression of which was increased in the muscle of sheep at 12 weeks of age compared to 3–5 days post-birth sheep [28]) and five differentially expressed lncRNAs and four other DEGs were selected to perform qRT-PCR analysis. The expression of them was consistent with the RNA-Seq results. Moreover, the RNA-Seq data of delta-like1 homologue gene (DLK1, which was enriched in fetal muscle [27]) and ras related dexamethasone induced 1 (RASD1, the expression of which was increased in the muscle of sheep at 12 weeks of age compared to 3–5 days post-birth sheep [28]) were also showed in Figure 5. The results confirmed the expression correlated well with the sequencing results and provided reliable validation for the sequencing data. The GenBank accession numbers and specific primers of each gene were listed in Table S1.

As lncRNAs could exert effects through cis-acting or trans-acting target genes, the neighboring (100 kb upstream or downstream) and/or complementary protein-coding genes of the differentially expressed lncRNAs from pairwise comparisons were predicted. Finally, a total of 201 target genes were obtained (Table S2). 

### 3.4. Bioinformatics Analysis of the Target Genes of the Differentially Expressed Long Non-Coding RNAs and Differentially Expressed Genes

**Figure 6:**
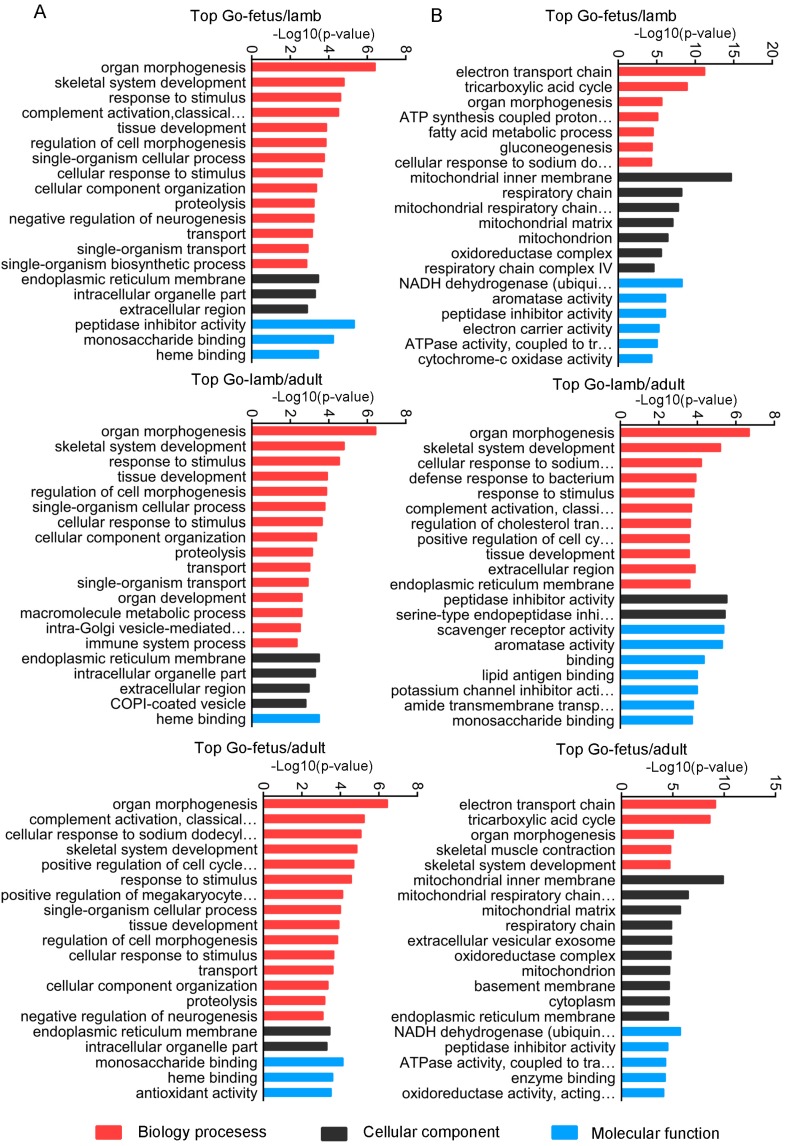
The top GO enrichment analyses of the target genes of the differentially expressed lncRNAs and DEGs in (control/experiment) fetus/lamb, lamb/adult and fetus/adult comparisons. (**A**) The top 20 GO terms enriched by the target genes of the differentially expressed lncRNAs in each comparison. (**B**) The top 20 GO terms enriched by DEGs in each comparison.

To gain more insights into the differentially expressed lncRNAs, the Nr, COG, GO and KEGG databases were used to annotate the target genes of the differentially expressed lncRNAs and DEGs (Tables S3 and S4). The overall functional annotation is described in Table 2. Based on the GO database, target genes and DEGs were assigned to biological processes, cellular components, and molecular function respectively. Moreover, organ morphogenesis, skeletal system development and response to stimulus, which all play vital roles in muscle growth, were identified as significantly enriched GO terms in all contrasts. The top 20 significant enriched GO terms for target genes and DEGs in each comparison are shown in Figure 6. 

**Figure 7:**
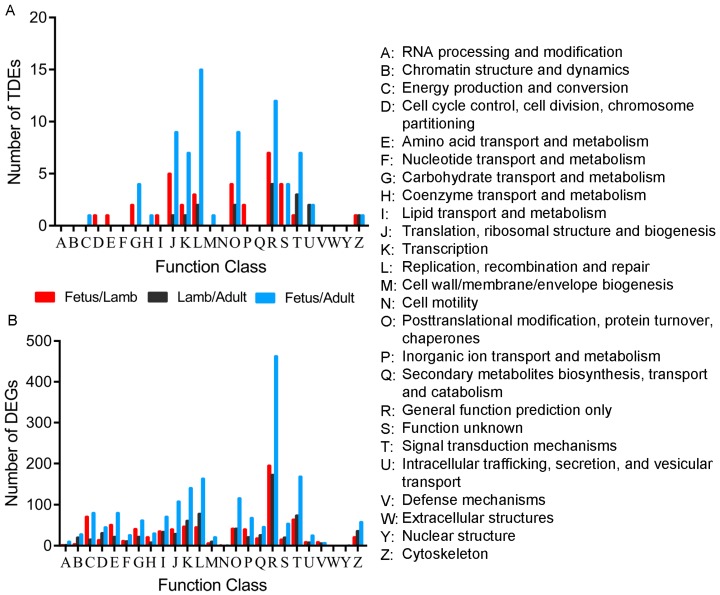
COG classification of all the target genes of the differentially expressed lncRNAs and DEGs during sheep muscle growth. (**A**) COG analysis for all the target genes of the differentially expressed lncRNAs. (**B**) COG analysis for all the DEGs.

Using the COG tools, the target genes of the differentially expressed lncRNAs and DEGs were functionally clustered into 17 and 24 classifications respectively. General function prediction only was the term for most of the target genes and DEGs. Moreover, the proportions of target genes and DEGs in fetus vs. lamb in amino acid transport and metabolism, carbohydrate transport and metabolism, as well as inorganic ion transport and metabolism, were higher than in the other two comparisons, suggesting the importance of these genes in the prenatal growth of muscle. In addition, higher proportions of signal transduction mechanisms and cytoskeleton were present in the comparison of lamb vs. adult than in the other two comparisons whether the analysis was for the target genes or DEGs, suggesting that these terms and their corresponding genes play vital roles in postnatal growth of muscle (Figure 7). 

According to the KEGG analysis, 21 (fetus vs. lamb), six (lamb vs. adult), and 51 (fetus vs. adult) target genes of lncRNAs were assigned to 40, 16, and 148 pathways, respectively. Although the number of the target genes of the differentially expressed lncRNAs in each comparison was low, pathways such as the mitogen-activated protein kinase (MAPK) signaling pathway, gap junction, calcium signaling pathway, insulin signaling pathway and regulation of actin cytoskeleton were included. Of these pathways, the MAPK signaling pathway was the most frequent pathway present in the fetus vs. lamb and fetus vs. adult comparisons, which inferred the pathway and its related lncRNAs potentially participated in regulation of the growth of sheep muscle. The enriched KEGG pathways with corrected *p*-value < 0.05 for the DEGs in each comparison, except for several disease related pathways, are listed in Table 3. The results showed that oxidative phosphorylation, carbon metabolism and cardiac muscle contraction were the first three pathways with the highest number of DEGs in the fetus vs. lamb and fetus vs. adult comparisons, which were not present in lamb vs. adult. Notably, pathways such as glycolysis/gluconeogenesis, fatty acid degradation, biosynthesis of amino acids and the peroxisome proliferator-activated receptor (PPAR) signaling pathway were independently detected in fetus vs. lamb, which proposed that DEGs in these pathways play crucial roles in the early stage of myofiber growth. Moreover, the phosphatidylinositol-3-kinase protein kinase B (PI3K-Akt) signaling pathway and steroid biosynthesis were present in the comparison of lamb vs. adult specifically, which enhanced their roles in postnatal muscle growth. Altogether, the differentially expressed lncRNAs and DEGs showed great potential in the regulation of muscle growth. 

### 3.5. Screening of Potential Functional Long Non-Coding RNAs Involved in Myofiber Growth 

**Figure 8:**
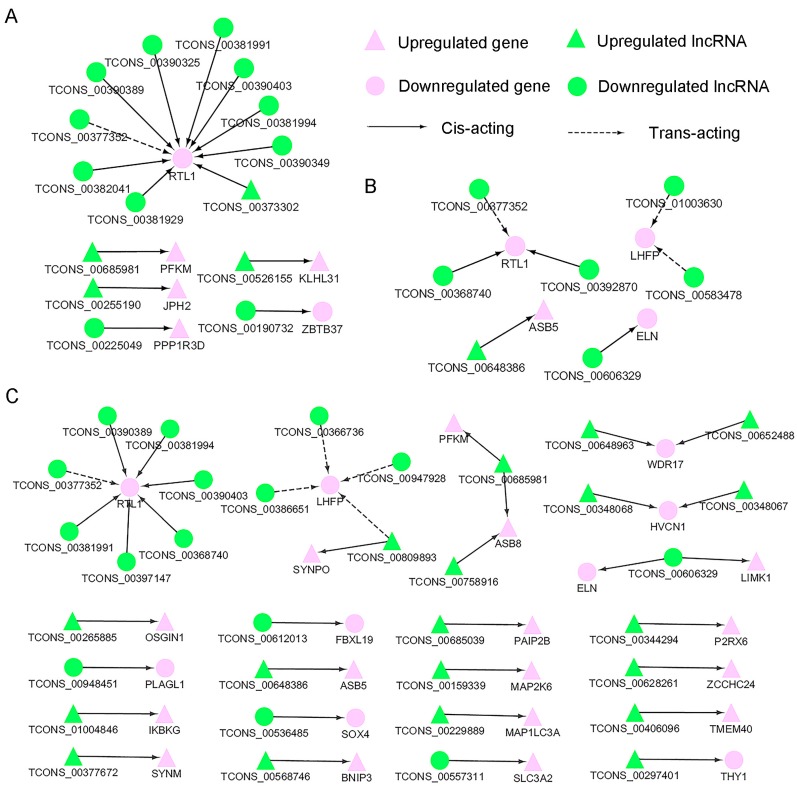
LncRNA–gene networks for (control/experiment) fetus/lamb, lamb/adult and fetus/adult comparisons. (**A**) LncRNA–gene network for the comparison of fetus/lamb. (**B**) LncRNA–gene network for the comparison of lamb/adult. (**C**) LncRNA–gene network for the comparison of fetus/adult. Differentially expressed lncRNAs and their corresponding differentially expressed cis- and trans-acting target genes were used to construct the lncRNA–gene interaction network. In this network, genes are displayed in purple while lncRNAs are displayed in green; upregulation is indicated with triangles while downregulation is indicated with circles. The cis-acting interactions are represented as solid lines, whereas the trans-acting interactions are represented as dashed lines. RTL1, retrotransposon-like 1; JPH2, junctophilin-2; ELN, elastin; LIMK1, LIM domain kinase 1; PFKM, phosphofructokinase, muscle; ASB8, ankyrin repeat and SOCS box containing 8; USP2, ubiquitin specific peptidase 2; ASB5, ankyrin repeat and SOCS box containing 5; WDR17, WD repeat domain 17; LHFP, lipoma HMGIC fusion partner; HVCN1, hydrogen voltage gated channel 1; KLHL31, kelch like family member 31; ZBTB37, zinc finger and BTB domain containing 37; PPP1R3D, protein phosphatase 1 regulatory subunit 3D; SYNPO, synaptopodin; OSGIN1, oxidative stress induced growth inhibitor 1; PLAGL1, PLAG1 like zinc finger 1; IKBKG, inhibitor of nuclear factor kappa B kinase subunit gamma; SYNM, synemin; FBXL19, F-box and leucine rich repeat protein 19; SOX4, SRY-box 4; BNIP3, BCL2 interacting protein 3; PAIP2B, poly(A) binding protein interacting protein 2B; MAP2K6, mitogen-activated protein kinase kinase 6; MAP1LC3A, microtubule associated protein 1 light chain 3 alpha; SLC3A2, solute carrier family 3 member 2; P2RX6, purinergic receptor P2X 6; ZCCHC24, zinc finger CCHC-type containing 24; TMEM40, transmembrane protein 40; THY1, Thy-1 cell surface antigen.

**Figure 9:**
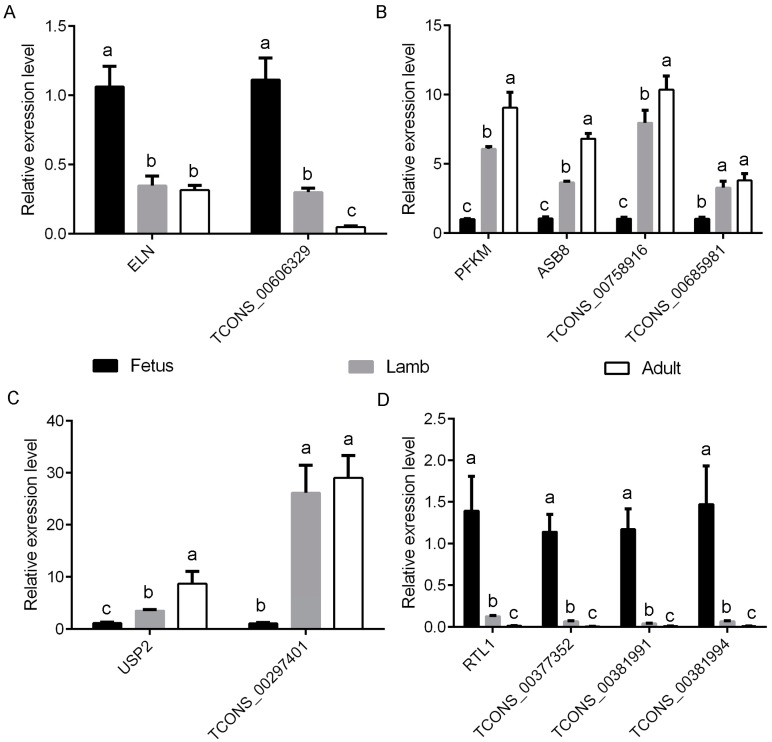
The verification of the expression level of the differentially expressed lncRNAs and the co-expressed target genes at fetus, lamb and adult stages measured by qRT-PCR. (**A**) The relative expression of TCONS_00606329 and target gene ELN. (**B**) The relative expression of TCONS_00758916 and TCONS_00685981 and the target genes PFKM and ASB8. (**C**) The relative expression of TCONS_00297401 and the target gene USP2. (**D**) The relative expression of TCONS_00377352, TCONS_00381991 and TCONS_00381994 and their target gene RTL1. The qRT-PCR data were represented as the mean ± SEM of three biological and technical replicates. Columns with different letters are significantly different at *p* < 0.05.

To further examine how lncRNAs cooperate with target genes to regulate muscle growth, co-expression analysis of the differentially expressed lncRNAs and the corresponding differentially expressed target genes was performed based on their FPKM. A total of 15 (fetus vs. lamb), seven (lamb vs. adult), and 37 (fetus vs. adult) significantly coregulated lncRNA–gene pairs were obtained. All three networks provide candidate lncRNAs related to muscle growth (Figure 8). Furthermore, the differentially expressed target genes that are directly involved in muscle development and their corresponding differentially expressed lncRNAs were classified. Consequently, eight biological processes from the GO analysis (muscle cell cellular homeostasis, muscle contraction, muscle organ development, skeletal muscle cell differentiation, skeletal system development, skeletal system morphogenesis, striated muscle cell development and organ development with corresponding 20 mRNAs and 41 lncRNAs were obtained, such as retrotransposon-like 1 (RTL1) and Junctophilin-2 (JPH2) (Table S5). The qRT-PCR analysis demonstrated that the expression of lncRNAs altered consistently with corresponding target genes during muscle growth, including TCONS_00606329 and its target gene elastin; TCONS_00758916 and TCONS_00685981 and their target genes phosphofructokinase, muscle and ankyrin repeat and SOCS box containing 8; TCONS_00297401 and the target gene ubiquitin specific peptidase 2; TCONS_00377352, TCONS_00381991 and TCONS_00381994 and their target gene RTL1 (Figure 9). These results further revealed the cooperative relationships of lncRNAs and their target genes. As the sequences and sites of these lncRNAs are incomplete from the sequencing analysis, further investigation must be performed in the future. 

## 4. Discussion

Accumulating evidence indicates the important roles of lncRNAs in muscle development of mice [12,29] , pigs [30], cows [26], goats [31] and humans [12]. However, transcriptome analysis of sheep muscle has been performed only at the fetal stage or one age without the inclusion of lncRNAs [32]. In this study, we performed strand-specific Ribo-Zero RNA-Seq, which detects poly(A)+ and poly(A)− RNAs from intact or fragmented RNA samples [33], to systematically identify sheep LD muscle lncRNAs across three time points. We investigated the sheep muscle lncRNAs from their transcript structure and expression patterns and further predicted the target genes of the lncRNAs to uncover their function. These results are discussed in detail and a more comprehensive network with potential functional lncRNAs is shown. To our knowledge, this study represents the first systematic genome-wide analysis of lncRNA in Hu sheep muscle, providing a valuable resource for functional lncRNAs associated with sheep muscle growth. 

In the present study, a total of 6924 lncRNAs were identified. Consistent with previous studies, most of the lncRNAs were found to transcribe near (100 kb) protein-coding genes, which implies that the lncRNAs have cooperative relationships with mRNAs. Moreover, similar to mRNAs, lncRNAs were widespread in chromosomes including 26 autosomes and the X-chromosome, which at least reflected the complexity and functional diversity of these lncRNAs. Notably, the proportion of lncRNAs in chromosome 18 was greater than the chromosome size proportion, which could be explained by the close relationship between chromosome 18 and muscle growth, as the well-known muscle growth regulators, the DLK1, CLPG and RTL1 were located in the chromosome. In addition, sheep muscle lncRNAs shared many typical properties with other mammalian lncRNAs, such as a relatively lower number of exons (mostly 2–5), a bigger exon size and lower expression levels compared to mRNAs [30,34]. These similarities supported that the potential lncRNAs identified in this study were reliable. However, despite the similarities, the difference between the number of muscle lncRNAs and skin lncRNAs might be attributed to tissue- and stage-specific characteristics. 

In this study, the expression of protein-coding genes and lncRNAs showed significant changes through the three developmental stages. Some were robustly expressed in the fetus stage, while others exerted effects in the lamb and adult stages. The qRT-PCR results further validated that the expression of the differentially expressed lncRNAs and DEGs was consistent with the sequencing data. As functional annotation is widely used to understand gene function, we attempted to uncover the roles of lncRNAs with dissection of their cis- and trans-acting target genes. GO analysis of the target genes of the differentially expressed lncRNAs and the DEGs showed that they are involved in several important bio-functions and processes such as organ morphogenesis, skeletal system development and response to stimulus. COG classification showed that amino acid transport and metabolism, carbohydrate transport and metabolism and inorganic ion transport and metabolism play vital roles in prenatal myofiber growth. In previous studies, amino acid (AAs), carbohydrates and inorganic ions were found to be essential in muscle growth. AAs are not only required for protein synthesis, but also act as signaling molecules in regulating muscle growth [35,36]. To maintain normal energy storage and consumption, carbohydrate transport and metabolism in skeletal muscle are indispensable. Inorganic ions, such as calcium, are essential in muscle plasticity and blocking of the calcium channel can lead to myofiber necrosis [37,38]. These findings emphasize the importance of these functional terms as well as their corresponding target genes and DEGs in the development of muscle, which further enhanced the accuracy of our prediction of key genes. Furthermore, the KEGG analysis confirmed that several differentially expressed lncRNAs in fetus vs. lamb and fetus vs. adult might be related to muscle biology. In addition, DEGs were associated with several signaling pathways related to muscle growth, such as the citrate cycle (TCA cycle) and the PI3K-Akt signaling pathway. The TCA cycle is involved in skeletal muscle fiber transition, which was reported in a previous study [39]. The PI3K-Akt signaling pathway is critical for protein synthesis and degradation in skeletal muscle, which is pivotal in counteracting muscle atrophy [40,41]. As a major metabolic tissue, it was not surprising that metabolic pathways, including pathways associated with fatty acid metabolism such as fatty acid degradation and carbohydrate metabolism processes (such as carbon metabolism and glycolysis/gluconeogenesis) were enriched. The bioinformatics results suggested that the genes and lncRNAs related to these regulatory processes showed significant changes during development, which means they may be considered potential candidate genes for further study of the molecular mechanisms of myofiber growth. However, how these genes cooperate with each other to exert their effects remains largely unknown. 

Therefore, we suggested that differentially expressed target genes as well as their corresponding differentially expressed lncRNAs might play a determinative role in the biofunction of sheep muscle. Thus we constructed lncRNA–gene interaction networks by integrating differentially expressed lncRNAs, differentially expressed target genes and their co–regulation relationships. According to the networks, some DEGs were previously reported to be regulators of muscle growth such as RTL1, which was predicted to be a target of multiple lncRNAs in all three contrasts. The ovine RTL1 (also known as PEG11) located in the DLK1 and the type III iodothyronine deiodinase gene (Dlk1-Dio3) imprinted locus, were reported to enhance muscle growth in mice. This result together with the ectopic expression of PEG11 in callipygian sheep strongly supports that this gene is responsible in muscular hypertrophy [42,43]. In the present study, the expression of abundant lncRNAs including maternally expressed gene 3 (MEG3/Gtl2), maternally expressed gene 8 (MEG8) and antisense RTL1 were correlated with the expression of RTL1. The coincidence of their expression was consistent with previous studies. However, although the expression of MEG3 and MEG8 was significantly altered by the mutation in CLPG, they did not display direct action on muscular hypertrophy [44,45]. Moreover, MEG3 was found to be expressed during neural development, which indicated that this gene might be implicated in the regulation of muscle performance [43,46]. Similar to RTL1, the antisense RTL1 is expressed at higher levels in skeletal muscle at the fetus stage than other stages, which might be attributed to the gene’s role in encoding several microRNAs that are key regulators of muscle development [47]. However, microRNAs that may contribute to phenotypic variation in skeletal muscle were not involved in this study [48]. In the future, integration analysis of microRNA, lncRNA and microRNA will be investigated, and the mechanism of muscle growth will be presented more clearly. Altogether, the positive relationship between RTL1 and multiple lncRNAs revealed that lncRNAs might target RTL1 via cis-acting to exert their control of the growth of sheep skeletal muscle. 

Apart from RTL1-associated lncRNAs, of the most interest, TCONS_00255190 (differentially expressed lncRNA presented specifically in the fetus vs. lamb network) caught our eyes for its predicted role in regulating JPH2, which is considered a critical regulator of T-tubule maturation in cardiac myocytes. Additionally, mutation in JPH2 can lead to dilated cardiomyopathy. Both results were interpreted as evidence of the regulatory role of JPH2 in skeletal muscle biology [49,50]. In the lamb vs. adult comparison, we noticed a unique lncRNA named TCONS_00583478 might target lipoma HMGIC fusion partner (LHFP), therefore, this lncRNA might be involved in muscle lipid metabolism. Further investigation needs to be performed to uncover their relationship in the future [51]. Another differentially expressed lncRNA presented in the lamb vs. adult and fetus vs. adult networks named TCONS_00606329 was predicted to target elastin, which is mainly responsible for tissue elasticity and plays vital roles in muscle biology [52,53,54]. This study underscores the potential importance of TCONS_00606329 in regulating muscle performance. According to the lncRNA-mRNA networks, phosphofructokinase, Muscle, ankyrin repeat and SOCS box-containing 5 gene and other genes were predicted to be targets of the differentially expressed lncRNAs. To the best of our knowledge, before this research, no report has connected them to lncRNA. Furthermore, the lncRNA-gene pairs directly involved in the muscle growth were classified including the inhibitor of kappa light polypeptide gene-TCONS_01004846, PLAG1 like zinc finger 1-TCONS_00948451. Taken together, these studies indicate that the lncRNAs identified in this study cooperate with their target DEGs to regulate muscle growth. 

## 5. Conclusions

In conclusion, the present data provide the first systematic description of lncRNA in the growth of sheep muscle. Using the Illumina platform, we screened a set of lncRNAs and genes related to muscle growth at three developmental stages in sheep. The lncRNAs identified in this study shared many properties with other mammalian lncRNAs. With reference to the GO, COG and KEGG databases, the target genes of differentially expressed lncRNAs and DEGs were annotated in multiple biological processes associated with muscle. Furthermore, the visual lncRNA–gene transcription regulatory networks generated in this study provide a valuable resource of candidate lncRNAs that could be utilized in the exploration of functional lncRNAs in sheep and thus may be translated to improve the growth of skeletal muscle. 

## Figures and Tables

**Table 1:** Summary of clean reads mapping to the *Ovis aries* reference genome in different stages.

Sample	Fetus	Lamb	Adult
Total Mapped Reads	65,578,070	65,591,958	71,241,551
Uniq Mapped Reads	60,846,465(92.76%)	60,372,363(92.06%)	66,521,969(93.43%)
Multiple Mapped Reads	4,731,605(7.24%)	5,219,595(7.94%)	4,719,582(6.57%)
Reads Map to ‘+’	34,126,308(52.04%)	34,153,600(52.07%)	36,918,137(51.82%)
Reads Map to ‘−’	31,451,762(47.96%)	31,438,358(47.93%)	34,323,414(48.18%)
Reads Map to exonic region	45.1%	53.8%	53.7%
Reads Map to intronic region	25.4%	19.6%	19.2%
Reads Map to intergenic region	29.5%	26.6%	27.1%

**Table 2:** The functional annotation of the target genes of the differentially expressed lncRNAs (targets) and differentially expressed genes (DEGs) using non-redundant protein sequence database (Nr), Clusters of Orthologous Groups of proteins (COG), Gene Ontology (GO) and Kyoto Encyclopedia of Genes and Genomes (KEGG)

#DEG Set (Control/Experiment)	Total Annotated		Nr		COG		GO		KEGG	
	Targets	DEGs	Targets	DEGs	Targets	DEGs	Targets	DEGs	Targets	DEGs
Fetus/Adult	150	3564	150	3564	57	1353	137	3317	83	2353
Fetus/Lamb	56	1480	56	1480	21	596	52	1376	37	972
Lamb/Adult	33	1536	33	1536	9	510	32	1436	20	997

**Table 3:** Enriched KEGG pathways of DEGs in (control/experiment) fetus/lamb, lamb/adult, and fetus/adult comparisons, respectively.

KEGG Pathway (KEGG Orthology (ko) ID)	Fetus/Lamb		Lamb/Adult		Fetus/Adult		All Unigenes	
	DEGs Number	%	DEGs Number	%	DEGs Number	%	Gene Number	%
1. Oxidative phosphorylation (ko00190)	74	12.67	0	0.00	72	5.26	164	2.11
2. Carbon metabolism (ko01200)	40	6.85	0	0.00	51	3.72	112	1.44
3. Cardiac muscle contraction (ko04260)	32	5.48	0	0.00	37	2.70	91	1.17
4. PI3K-Akt signaling pathway (ko04151)	0	0.00	50	8.55	0	0.00	374	4.81
5. Adrenergic signaling in cardiomyocytes (ko04261)	0	0.00	0	0.00	48	3.50	159	2.05
6. Citrate cycle (TCA cycle) (ko00020)	22	3.77	0	0.00	22	1.61	34	0.44
7. Fatty acid metabolism (ko01212)	16	2.74	0	0.00	21	1.53	51	0.66
8. Pyruvate metabolism (ko00620)	16	2.74	0	0.00	20	1.46	42	0.54
9. Focal adhesion (ko04510)	0	0.00	33	5.64	0	0.00	224	2.88
10. Cell cycle (ko04110)	0	0.00	27	4.62	0	0.00	148	1.90
11. Propanoate metabolism (ko00640)	10	1.71	0	0.00	15	1.09	27	0.35
12. Proteasome (ko03050)	0	0.00	0	0.00	22	1.61	47	0.60
13. Biosynthesis of amino acids (ko01230)	21	3.60	0	0.00	0	0.00	80	1.03
14. Glycolysis/Gluconeogenesis (ko00010)	19	3.25	0	0.00	0	0.00	70	0.90
15. PPAR signaling pathway (ko03320)	19	3.25	0	0.00	0	0.00	70	0.90
16. Fatty acid degradation(ko00071)	14	2.40	0	0.00	0	0.00	44	0.57
17. Alanine, aspartate and glutamate metabolism (ko00250)	13	2.23	0	0.00	0	0.00	40	0.51
18. 2-Oxocarboxylic acid metabolism (ko01210)	11	1.88	0	0.00	0	0.00	18	0.23
19. Nicotinate and nicotinamide metabolism (ko00760)	10	1.71	0	0.00	0	0.00	29	0.37
20. Steroid biosynthesis (ko00100)	0	0.00	8	1.37	0	0.00	22	0.28

## Supplementary Materials

The following are available online at www.mdpi.com/2073-4425/8/8/191/s1. Table S1: Primers for qRT-PCR, Table S2: Differentially expressed lncRNAs and their corresponding target genes in fetus vs. lamb, lamb vs. adult and fetus vs. adult comparisons, respectively, Table S3: Annotation of the target genes of differentially expressed lncRNAs in fetus vs. lamb, lamb vs. adult and fetus vs. adult comparisons, respectively, Table S4: Annotation of DEGs in fetus vs. lamb, lamb vs. adult and fetus vs. adult comparisons, respectively, Table S5: Muscle growth associated differentially expressed lncRNAs and their target genes.
