# Supplementary material for: Genome-Wide Analysis Reveals Extensive Changes in LncRNAs during Skeletal Muscle Development in Hu Sheep
Source: Genes (Basel). 2017 Aug 1;8(8):191. doi: 10.3390/genes8080191 (PMC5575655; doi:10.3390/genes8080191)
Supplement: Supplementary file 1 [file genes-08-00191-s001.zip › Supplementary/Table S1.docx]

**Supplementary Table S1 Primers for qRT-PCR**

| Genes and Reference Sequences (Gen bank no.) | Primer Sequences (5’ to 3’) | Size of PCR Product, bp |
| --- | --- | --- |
| HPRT1  （XM_015105023.1） | F: CCATCACATTGTGGCCCTCT | 163 |
|  | R: GTCACCTGTTGACTGGTCGT |  |
| TCONS_00138959 | F: GACGACCTCGAGCAAGTCAT | 207 |
|  | R:ACTGGGCACAGAGTAACTGC |  |
| TCONS_00588845 | F: ACAGCACTAATGCCATGCCT | 152 |
|  | R: CACACTGTGGCATTCCTGTTAC |  |
| TCONS_00123794- | F: GCACGAAAGCATCTCACGTTT | 75 |
|  | R: GTCTGTCTCTGTGTGGCAGC |  |
| TCONS_00397147 | F: ACTTATGGTGGGTGTGCCAA | 169 |
|  | R: CAACCAGAGAGCATGTCCGT |  |
| TCONS_00648963- | F: CAAGCTTGCGGGAGTAAAGC | 100 |
|  | R: AGCCTGTTGGTTGGGAGTTC |  |
| GOT2-  (XM_012156902.2) | F: CATTGGGGGACTGGCTGAAT | 103 |
|  | R: AATGGTCTGCACGGTGACAT |  |
| MKNK1  (XM_015091954.1) | F: CACAGTGTGCCAGAACAAGC | 138 |
|  | R: AGTCTCTGCTTCGCATCTCG |  |
| PPP1R16B  (XM_015099883.1) | F: CATAGCTGGAGCCAACGGAT | 141 |
|  | R: AACAGCTCTGCCATCTGCAT |  |
| SMOX  (XM_012188709.2) | F: GCAACAGCCTACGGTTTGTG | 114 |
|  | R: GCTCAGGAGGGTAGAGGACA |  |
| MYOG-  (NM_001174109.1) | F: GCAGCGCCATCCAGTACATA | 144 |
|  | R: GACTGCAGGAGGCACTATGG |  |
| MYH7 | F: GTTTGAAAAGCCAAGCCGC | 123 |
| (XM_012129251.1) | R: TGAGGTCAAAAGGCCTGGTC |  |
| TCONS_00606329 | F: GGGGATATAGCTCCCCAGATCA | 187 |
|  | R: GCCCTGCCCATCTCTATGTG |  |
| ELN  ( XM_015103908.1) | F: GTGCCCCAGGAGCAATACCA | 122 |
|  | R: CAGGGCCCCTTCCATGAGATAA |  |
| TCONS_00758916 | F: CCACCAAACAAGGTCCAGAA | 142 |
|  | R: CGACAATCCAGGGTTCTTCCT |  |
| TCONS_00685981 | F: TGTCATCTCAGACTGTCACACC | 256 |
|  | R: TGGGGTGTTTCAAGGAACAGA |  |
| PFKM,  (XM_004006406.3) | F: GCAGTAATGGCGCTTTTGGA | 185 |
|  | R: ATTCATGCCCGCAGCCG |  |
| ASB8  XM_012174381.2 | F:TGCTCTCCATGTAGTAGAAGAGAT  R: AGAGCCTACCAAGCCCACTA | 116 |
| TCONS_00297401 | F: CAACGGCCCTGTGAATTTCG | 152 |
|  | R:GATTACCCGGCCCAAGTCAG |  |
| USP2  (XM_012095607.2) | ATATCCCTCAGCCTCTTCCCT | 200 |
|  | CCAACAGCTGGGGAGTAGAC |  |
| TCONS_00377352 | AAATGAGCCATGGACCAGCC | 197 |
|  | TGTCTGCAGTAGCCACTCATT |  |
| TCONS_00381991 | TGTTGCCCATAGTTCAGTGGT | 296 |
|  | TCGTTCAGGACTCTACCCGA |  |
| RTL1  (XM_015102054.1) | GGCTCTCCGACAGAGGTGAT | 171 |
|  | TGGATGAAGGCTTGATGGCT |  |
| TCONS_00381994 | GTGGTCTTGGGTTGAATGGC | 212 |
|  | TGACACAGCCAACTCTCATGT |  |
